# Exploring Regulation Genes Involved in the Expression of L-Amino Acid Oxidase in *Pseudoalteromonas* sp. Rf-1

**DOI:** 10.1371/journal.pone.0122741

**Published:** 2015-03-27

**Authors:** Zhiliang Yu, Ju Wang, Jianxun Lin, Minyan Zhao, Juanping Qiu

**Affiliations:** 1 College of Biological and Environmental Engineering, Zhejiang University of Technology, Hangzhou 310014, China; 2 Department of Electrical Engineering, Columbia University, New York 10027, United States of America

## Abstract

Bacterial L-amino acid oxidase (LAAO) is believed to play important biological and ecological roles in marine niches, thus attracting increasing attention to understand the regulation mechanisms underlying its production. In this study, we investigated genes involved in LAAO production in marine bacterium *Pseudoalteromonas* sp. Rf-1 using transposon mutagenesis. Of more than 4,000 mutants screened, 15 mutants showed significant changes in LAAO activity. Desired transposon insertion was confirmed in 12 mutants, in which disrupted genes and corresponding functionswere identified. Analysis of LAAO activity and *lao* gene expression revealed that GntR family transcriptional regulator, methylase, non-ribosomal peptide synthetase, TonB-dependent heme-receptor family, Na^+^/H^+^ antiporter and related arsenite permease, N-acetyltransferase GCN5, Ketol-acid reductoisomerase and SAM-dependent methytransferase, and their coding genes may be involved in either upregulation or downregulation pathway at transcriptional, posttranscriptional, translational and/or posttranslational level. The *nhaD* and *sdmT* genes were separately complemented into the corresponding mutants with abolished LAAO-activity. The complementation of either gene can restore LAAO activity and *lao* gene expression, demonstrating their regulatory role in LAAO biosynthesis. This study provides, for the first time, insights into the molecular mechanisms regulating LAAO production in *Pseudoalteromonas* sp. Rf-1, which is important to better understand biological and ecological roles of LAAO.

## Introduction

L-Amino acid oxidase (LAAO; EC 1.4.3.2) is usually a flavin adenine dinucleotide (FAD)-containing homodimeric protein, which functions in stereospecific oxidative deamination of L-amino acids to the corresponding a-keto acids with release of NH_4_^+^ and H_2_O_2_ [1]. It plays important biological roles, such as apoptosis [2], cytotoxicity [3], edema [4], hemolysis, hemorrhage [5], inducing or inhibiting platelet aggregation [6], and parasite-killing and antimicrobial activities [7]. Most of its biological functions are associated with the produced H_2_O_2_ which is toxic to most organisms [8, 9]. Therefore, LAAO is considered as a toxic protein and characterization of the mechanisms controlling its expression is very important for understanding not only its biological but also ecological roles.

The enzymatic and physiochemical properties, biological function and structure of LAAO as well as its cloning and heterologous expression have been intensively investigated [9]. However, studies on unraveling the mechanism regulating LAAO expression are limited, where only a few genes controlling its transcription and translation have been explored. Previously, it was found that LAAO activity in *Neurospora crassa* can be induced by L-Phe, D-Phe, ATP and cycloheximide. Those inducing agents can regulate the *lao* gene expression at transcriptional level [10]. However, the regulation genes were undefined. More recently, a spontaneous mutant of *N*. *crassa gln-1br8* with deficiency in glutamine synthetase β polypeptide was confirmed to have higher LAAO activity [11]. Again, the relevant genes for regulation were unexplored. It was also reported that LAAO activity in *N*. *crassa* can be induced through addition of L-amino acids to nitrogen-starved cultures as well as addition of protein synthesis inhibitors or D-amino acids [12, 13]. LAAO expression was regulated by NIT2 and the *nmr* gene product at transcriptional level [14]. Besides LAAO in *N*. *crassa*, LAAO from *Marinomonas mediterranea* was found to be regulated by hybrid sensor histidine kinase PpoS at transcriptional level [15]. Two genes of *lodAB* operon were found to be necessary for LAAO expression in *M*. *mediterranea* [16].

LAAO is widely found in marine *Pseudoalteromonas* spp. [17–19] and plays an important role in dispersal and colonization across a range of Gram-negative marine bacteria, such as *Pseudoalteromonas tunicate* [20], suggesting its important ecological function in marine environment. Recently, a yellow-pigmented marine bacterium *Pseudoalteromonas* sp. B-3 was isolated to produce LAAO [21, 22]. In this study, to explore the genes involved in regulating LAAO activity, a red-pigmented spontaneous mutant strain of *Pseudoalteromonas* sp. B-3 resistant to rifampicin, designated as *Pseudoalteromonas* sp. Rf-1, was selected. The transposon mutagenesis [23] was used to construct a mutant library of strain Rf-1 and the target mutants with altered LAAO activity were screened using Prussian blue agar assay method [22]. Based on genetic and enzymatic analysis, several regulation genes were identified. Our results shed some lights into the molecular mechanisms regulating LAAO activity in this marine bacterium across the natural environment.

## Results

### Mutant screening of LAAO activity

**Fig 1:**
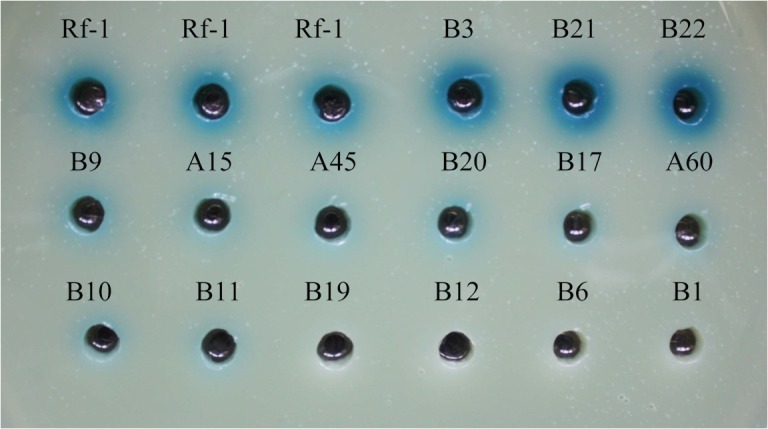
Measurement of LAAO activity from the LAAO-altered or -deficient mutants using Prussian blue agar assay [22]. 50 μl of culture supernatant of individual mutant or wild-type strain Rf-1 was added into the punched circular hole with a diameter of 6 mm in the Prussian blue agar assay plate for color development from Berlin green to blue caused by H_2_O_2_ produced by LAAO activity. The diameter of the resultant blue halo is exponentially associated with the LAAO activity. Compared with wild-type strain, the mutants B3, B21 and B22 had the increased LAAO activities, and the mutants B9, A15, A45, B20, B17, A60, B10 and B11 yielded the decreased LAAO activities, while the mutants B19, B12, B6 and B1 were deficient in LAAO activity.

To explore the genes regulating LAAO activity in *Pseudoalteromonas* sp. Rf-1, plasmid pLOF/Km carrying a mini-Tn*10* [24] was used to generate a mutant library of strain Rf-1. Antibiotics of rifampicin (Rif) and kanamycin (Km) were used to repress *Escherichia coli* with plasmid pLOF/Km and wild type strain Rf-1, respectively. After transposon mutagenesis [23, 25], approximately 4,000 mutants appeared on marine medium (MM) plate with Rif and Km. Then, culture supernatant of individual mutant was subjected to LAAO-activity screening with Prussian blue agar assay [22]. Altered LAAO activity was detected in 300 mutants,(data not shown) in which 15 mutants displayed significant altered or abolished LAAO activity (Fig. 1). As shown in Table 1, compared to wild-type strain, 3 mutants (B3, B21 and B22) gave increased LAAO activity, 8 mutants (B9, A15, A45, B20, B17, A60, B10 and B11) yielded decreased LAAO activities, while the other 4 mutants (B19, B12, B6 and B1) showed null LAAO activity. The statistical analysis showed that the mean difference of blue halo diameter or LAAO activity between mutant and wild type was all extremely significant (P<0.001) (Table 1). Therefore, these 15 mutants were selected for further investigation.

**Table 1:** Comparison of LAAO activity between mutant and wild type.

Strains	Prussian blue halo		LAAO	
	Diameters (cm)^a^	P^b^	Activity^c^	P
Rf-1	1.10±0.01	/	0.50	/
B3	1.20±0.03	***	1.87	***
B21	1.25±0.02	***	2.35	***
B22	1.25±0.02	***	2.35	***
B9	0.75±0.01	***	0.13	***
A15	0.75±0.01	***	0.13	***
A45	0.75±0.01	***	0.13	***
B20	0.75±0.02	***	0.13	***
B17	0.75±0.03	***	0.13	***
A60	0.70±0.02	***	0.10	***
B10	0.75±0.02	***	0.13	***
B11	0.75±0.01	***	0.13	***
B19	0.60±0	***	0	***
B12	0.60±0	***	0	***
B6	0.60±0	***	0	***
B1	0.60±0	***	0	***

^a^ All the diameters of Prussian blue halos were achieved based on triplicate experiments.

^b^ Significance of mean difference of Prussian blue halo diameter or LAAO activity between mutant and wild type was statistically analyzed by ANOVA; “***” means extremely significant (P<0.001).

^c^ LAAO activity was calculated on the basis of the Prussian blue halo diameter [22].

### Molecular confirmation of transposon insertion in mutants

**Fig 2:**
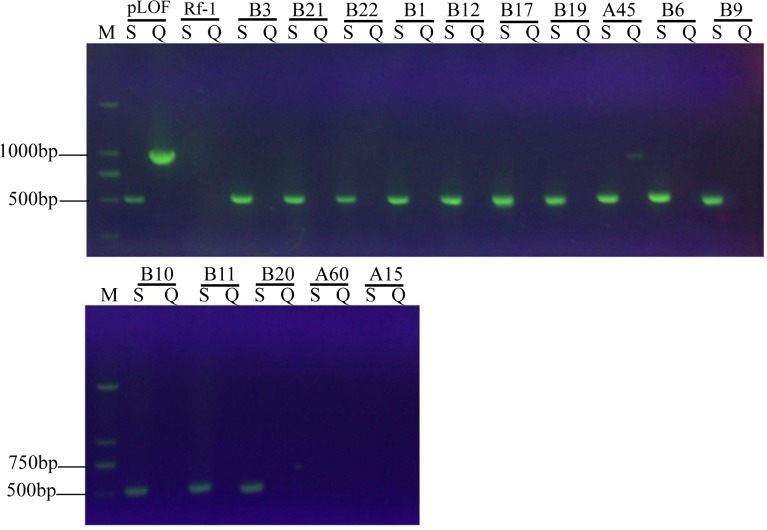
PCR detection of insertion of mini-Tn*10* together with kanamycin resistance marker (IS-km-IS region) in LAAO-altered mutants. S: PCR using primers Tn10KAP3 and S1; Q: PCR using primers DTn10AP3 and Q2; pLOF: PCR using plasmid pLOF/Km as template (positive control); Rf-1: PCR using genomic DNA of wild type strain Rf-1 as template (negative control); B3, B21, B1, B12, B19, A45, B6, B9, B10, B11, B20, A60 and A15: PCR reaction using genomic DNA from the corresponding mutant as template.

Two primer pairs were used to confirm the correct insertion of mini-Tn*10* gene along with Km resistance marker gene (IS-km-IS) in the 15 mutants from above. One pair was Tn10KAP3 (5’-CATTTGATGCTCGATGAGTTTTTCT-3’) and S1 (5’-TTGGTAAAAATCATTAAGTTAAGGT-3’), which were both specific to IS-km-IS region; the other pair was DTn10AP3 (5’-CGTTGCGCTGCCCGGATTACAGCCG-3’) and Q2 (5’-AAACGATGCCCATTTTGTTGATTAT-3’), which were specific to IS-km-IS and pLOF vector region (outside of IS-km-IS), respectively. The first primer pair was used to amplify a 506bp fragment, while the second one was use to amplify a 964bp fragment. As shown in Fig. 2, positive control of pLOF/Km yielded both fragments, while negative control (wild type strain Rf-1) gave neither. Mutants A60 and A15 exhibited neither fragment, suggesting unclear mutation or failure of PCR amplification; mutant A45 unexpectedly displayed both fragments, probably due to insertion of extra piece from pLOF/Km vector or even whole pLOF/Km. On the other hand, all the remaining 12 mutants expectedly gave only one fragment (506 bp) from primer pair of Tn10KAP3 and S1, but not the other one (964 bp) from primer pair of DTn10AP3 and Q2. All these results indicated that the correct insertion of IS-km-IS took place probably only in 12 mutants (B3, B21, B22, B9, B10, B11, B17, B20, B1, B6, B12 and B19) which were used in the following experiments.

### Quantitative real-time PCR (qRT-PCR) analysis of the differential expression of *lao* gene

**Fig 3:**
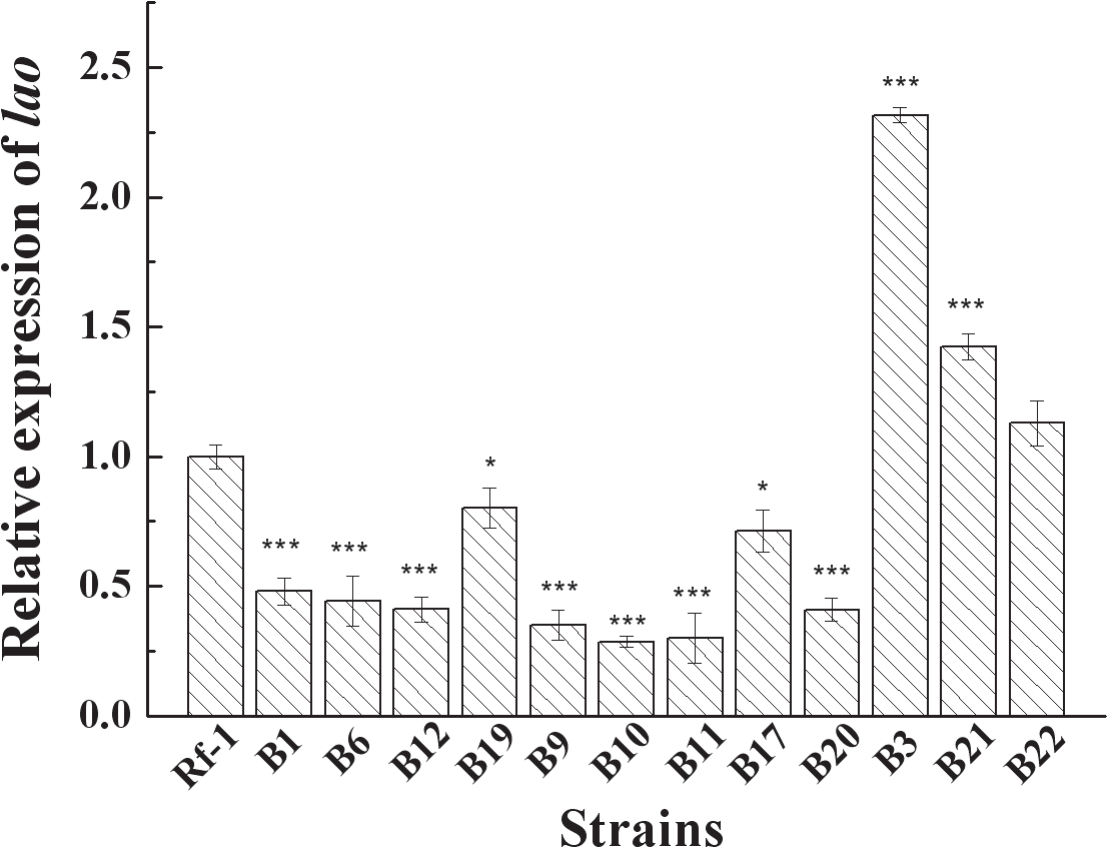
*lao* gene expression in mutants and wild-type strain Rf-1. Mean difference of relative expression of *lao* gene between mutant and wild type was statistically analyzed by ANOVA (S1 Dataset and S2 Dataset). “*” and “***” represent significant (P<0.05) and extremely significant (P<0.001), respectively.

The expression of *lao* gene in 12 mutants identified above and wild-type strain was measured by qRT-PCR [26]. The results in Fig. 3 showed that all the strains yielded the expression of *lao* gene. Compared to wild-type strain Rf-1, 3 mutants (B3, B21, and B22) had increased expression of *lao* gene, while the other 9 gave decreased expression. Overall, the qRT-PCR results showed the same tendency as LAAO enzymatic activity in the different mutants. In mutants with null activity, the qRT-PCR gave also lower levels of mRNA, while in mutants with higher LAAO activity it is possible to see higher mRNA levels (Fig. 1 and Table 1). Notably, the mutants (B1, B6, B12 and B19) with abolished LAAO activity still exhibited *lao* gene expression. Statistical analysis indicated that the mean difference of relative *lao* gene expression between mutant and wild type was significant (P<0.05) in mutants B19 and B17, and extremely significant (P<0.001) in mutants B1, B6, B12, B9, B10, B11, B20, B3 and B21, but not significant (P>0.05) in mutant B22. However, regarding LAAO activity, the mean differences between mutants and wild type all were extremely significant (P<0.001). Thus, all these findings indicated that LAAO activity and the corresponding *lao* gene expression are probably regulated by the disrupted genes in different mutants at different levels.

### Characterization of the genes disrupted by transposon in mutants

The DNA region flanking inserted transposon was sequenced for identification of the disrupted gene in each mutant. Typically, a DNA sequence with around 500–1200 bp for each mutant was obtained through hiTAIL-PCR [27]. As shown in Table 2, most of the sequences in mutants could match with the genes from the same genus, *Pseudoalteromonas rubra* ATCC 29570, with the exception that mutant B10 showed the highest similarity (68%) to a gene coding for TonB-dependent heme receptor family in *Alcanivorax dieselolei* B5. The disrupted gene resulting in increase of LAAO activity in mutant B22 showed the highest identity (72%) to a gene encoding GntR family transcriptional regulator. The possible corresponding genes resulting in decrease of LAAO activity in mutants B20, B17 and B10 had the closest similarity to the genes coding for methylase (80%), non-ribosomal peptide synthetase (82%) and TonB-dependent heme receptor family (68%), respectively. Interestingly, the disrupted genes in mutants B1, B6, B12 and B19 with abolished LAAO activity had the highest similarity to those coding for SAM-dependent methytransferase (80%), Ketol-acid reductoisomerase (80%), N-acetyltransferase GCN5 (80%), and Na^+^/H^+^ antiporter NhaD and related arsenite permease (81%), respectively. Besides, the disrupted genes in mutants B3 and B9 yielded no any similar gene in databases and the ones in mutants B21 and B11 all matched with the unannotated sequences. Therefore, we did not try to propose the function of these 4 genes. All the characterized gene sequences have been deposited in GenBank and the corresponding access no. was included in Table 2.

**Table 2:** Length of sequenced region and the closest match of the transposon-disrupted genes in the mutants with altered LAAO activity.

LAAO activity	Mutant	Length of sequence (bp)	Access No. in GenBank	Closest match in GenBank, ENA or Prosite		
				Gene function	Similarity (%)	Source organism
Increased	B3	649	KP234240	Unmatched	<30%	/
	B21	194	KP234241	Unannotated	89	*P*. *rubra* ATCC 29570
	B22	870	KP234242	GntR family transcriptional regulator (GntR)	72	*P*.* rubra* ATCC 29570
Decreased	B9	596	KP234243	Unmatched	<30%	*/*
	B20	1109	KP234244	Methylase	80	*P*. *rubra* ATCC 29570
	B17	587	KP234245	Non-ribosomal peptide synthetase (NrpS)	82	*P*. *rubra* ATCC 29570
	B10	785	KP234246	TonB-dependent heme receptor family (TdhR)	68	*Alcanivorax dieselolei* B5
	B11	595	KP234247	Unannotated	88	*P*. *rubra* ATCC 29570
Deficient	B19	685	KP234248	Na^+^/H^+^ antiporter NhaD and related arsenite permease (NhaD)	81	*P*. *rubra* ATCC 29570
	B12	786	KP234249	N-acetyltransferase GCN5 (Nat5)	80	*P*. *rubra* ATCC 29570
	B6	972	KP234250	Ketol-acid reductoisomerase (KarI)	80	*P*. *rubra* ATCC 29570
	B1	1120	KP234251	SAM-dependent methytransferase (SdmT)	80	*P*. *rubra* ATCC 29570

### Complementation of the disrupted genes in mutants with abolished LAAO-activity

**Fig 4:**
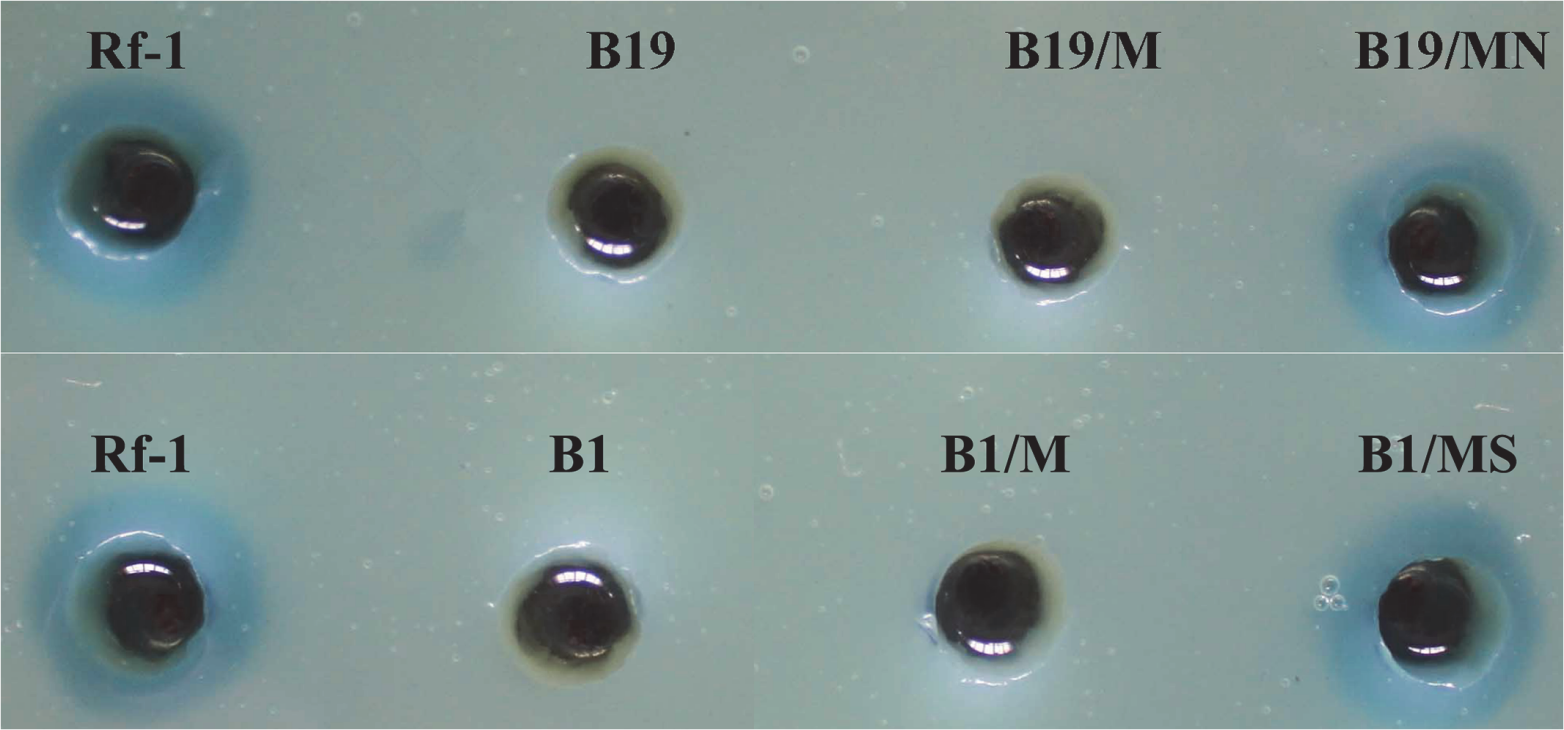
LAAO activity of wild type strain, mutants and the corresponding complemented strains. Rf-1: wild-type; B19: *nhaD* gene-disrupted Rf-1; B19/M: mutant B19 complemented with blank vector pBBR1MCS-5; B19/MN: mutant B19 complemented with recombinant plasmid pBBR1MCS-5/*nhaD*; B1: *sdmT* gene-disrupted Rf-1; B1/M: mutant B1 complemented with blank vector pBBR1MCS-5; B1/MS: mutant B1 complemented with recombinant plasmid pBBR1MCS-5/*sdmT*.

The transposon-disrupted genes in the 4 mutants with null LAAO-activity (B1, B6, B12 and B19) were identified as *sdmT*, *karI*, *nat5* and, *nhaD* with their corresponding functions displayed in Table 2. To further confirm that these identified genes were involved in regulation of LAAO biosynthesis, we tried to perform gene complementation in each mutant. We retrieved the full gene sequences of *sdmT* (access no. KP191657), *karI* (access no. KP191658) and *nhaD* (access no. KP191659), with length of 594 bp, 1473 bp and 1455 bp, respectively. However, the retrieval of full *nat5* gene failed, even after extensive optimization procedure on hiTAIL-PCR condition. Subsequently, the 3 successfully retrieved genes were cloned into vector pBBR1MCS-5 [28] to form pBBR1MCS-5/*sdmT*, pBBR1MCS-5/*nhaD* and pBBR1MCS-5/*karI*, respectively. The pBBR1MCS-5/*sdmT* and pBBR1MCS-5/*nhaD* were successfully transformed into mutants B1 and B19, respectively, with assistance of plasmid pBR2013 [29] in *E*. *coli* DH5α. However, the transformation of pBBR1MCS-5/*karI* into mutant B6 failed. Prussian blue agar assay in Fig. 4 showed that the two complemented strains (complons), B1/MS and B19/MN yielded the Prussian blue halos with comparable diameter to wild-type strain Rf-1, indicating that LAAO activity was nearly completely recovered. Accordingly, our data demonstrated that both *sdmT* gene and *nhaD* gene are involved in controlling LAAO biosynthesis in *Pseudoalteromonas* sp. Rf-1.

**Fig 5:**
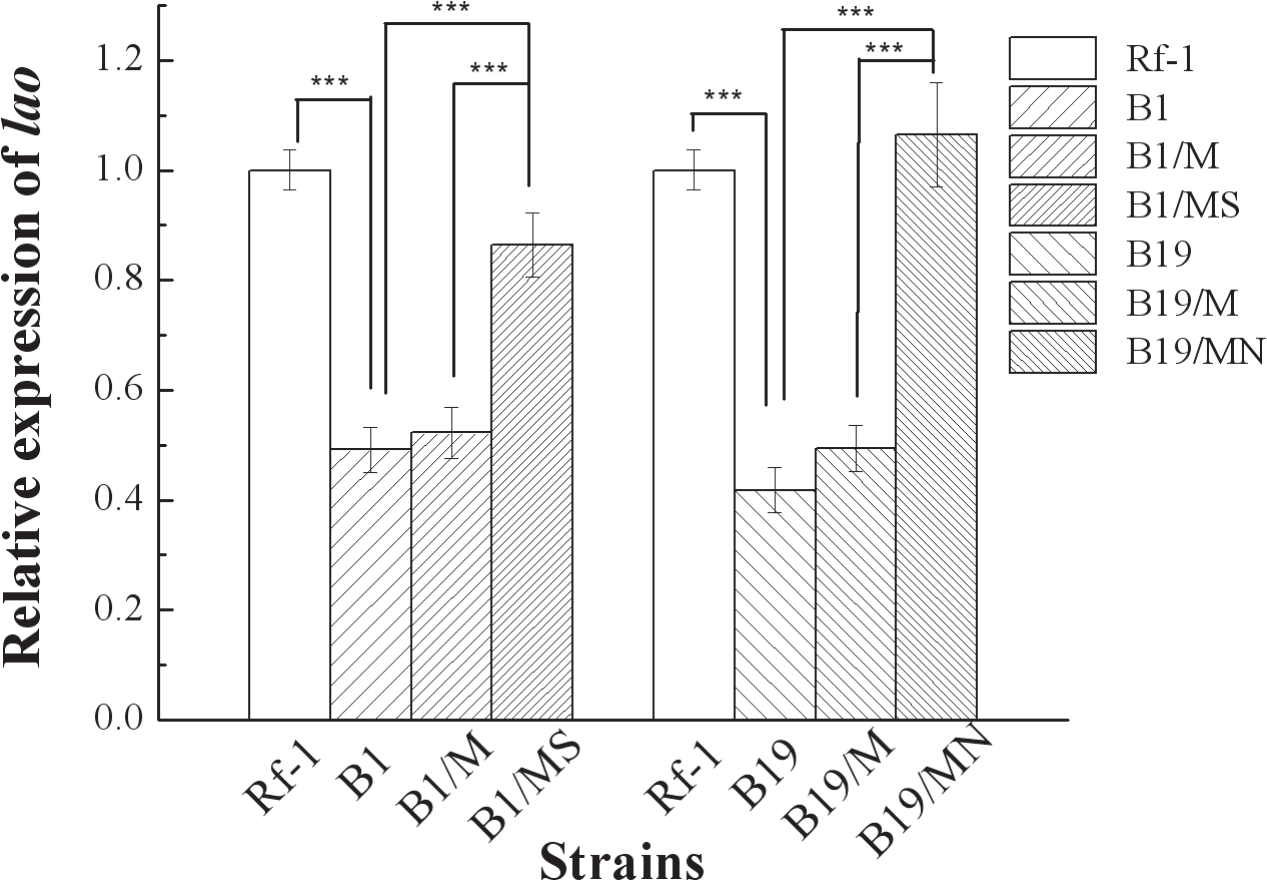
Relative expression level of *lao* gene in different strains determined by qRT-PCR [26]. Rf-1: wild type; B1 and B19: two mutants of wild type Rf-1 with transposon-inserted *sdmT* gene and *nhaD* gene, respectively; B1/M and B19/M: both mutant B1 and mutant B19 with blank delivery vector pBBR1MCS-5; B1/MS and B19/MN: mutant B1 and mutant B19 with complementary plasmid pBBR1MCS-5/*sdmT* carrying entire gene of *sdmT* and plasmid pBBR1MCS-5/*nhaD* carrying entire gene *nhaD*, respectively. Mean difference of relative expression of *lao* gene between different strains was statistically analyzed by ANOVA (S3 Dataset and S4 Dataset). “***” is extremely significant (P<0.001).

As shown in Fig. 5, the relative expression level of *lao* gene in either mutant B1 or mutant B19 was extremely significantly (P<0.001) decreased, compared to that in wild-type Rf-1. The complons B1/MS and B19/MN resulted from the complementation of *sdmT* gene and *nhaD* gene in B1 and B19, respectively, exhibited extremely significant (P<0.001) increase of *lao* gene expression, as compared to original mutants. In addition, their *lao* gene expression levels were comparable to that in wild-type Rf-1 (no significance, P>0.05), suggesting that the complementation of transposon-inserted genes can almost completely restore *lao* gene expression. The negative control using complons B1/M and B19/M with blank complementation lack of gene of interest displayed similar expression level as original mutants (no significance, P>0.05). Taken together, we concluded that both *sdmT* gene and *nhaD* gene upregulate the *lao* gene expression at transcriptional level in *Pseudoalteromonas* sp. Rf-1. Moreover, considering that the disruption of *sdmT* gene and *nhaD* gene resulted in completely undetectable LAAO activity, but only partial decrease of *lao* gene expression, we proposed that both genes also regulated LAAO biosynthesis probably at posttranscriptional level in *Pseudoalteromonas* sp. Rf-1.

## Discussion

Species of the genus *Pseudoalteromonas*, in particular color-pigmented ones, are generally found to be associated with marine eukaryotes and have antibacterial, algicidal, antifungal, or antiviral activity. Several *Pseudoalteromonas* isolates specifically deter the settlement of common marine fouling organisms. Thus, *Pseudoalteromonas* cells are advantageous in their contest for nutrients, space and colonization of surfaces, and are protected against predators grazing at surfaces [30, 31]. LAAO is believed to be associated with *Pseudoalteromonas’* biological and ecological roles in marine environment [30, 31]. Therefore, a thorough understanding of mechanisms controlling LAAO production is extremely important and is attracting a great number of interests.

Despite the prevalent application of mutagenesis technique, such as transposon mutagenesis, in mutant screening since the past decade, applying the detection method cheaply and efficiently for large-scale screening of mutants with different LAAO activities has proven challenging [32]. To date, very little is known about the regulation mechanism underlying LAAO transcription and production in marine bacteria. Previously, Prussian blue agar assay method has been developed with high efficiency and convenience, holding a promising application as a sensitive procedure for differentiating the mutants from a mutant library with altered LAAO activity [22]. In this study, over 4,000 mutants with Rif and Km resistance were isolated and screened using Prussian blue agar assay, where only about 300 (7.5%) mutants in total were found to have altered LAAO activity. Among them, more than 95% mutants only had slight changes in LAAO activity, resulting in the difficulty for clear differentiation of mutants, which in the future could be overcome by using ferric-xylenol orange formation (Fe^Ⅲ^-XO) agar assay method with higher sensitivity [32, 33] for detecting LAAO activity.

Nevertheless, the combination of mini-Tn*10* mutagenesis with Prussian blue agar assay allowed us to successfully identify several genes potentially controlling LAAO production in *Pseudoalteromonas* sp. Rf-1. Those identified genes could be grouped into two categories, positive regulator and negative regulator, based on the change of LAAO activity in mutants listed in Table 2.

The only identified negative regulator is GntR family transcriptional regulator that was found in mutant B22. The GntR family is one of the most abundant and widely distributed groups of transcriptional regulators in bacteria [34]. It was reported that the *gntR* gene acts to repress transcription of itself as well as a series of genes immediately adjacent to it [35, 36]. Mutation of *gntR* gene resulted in increased LAAO activity, indicating a downregulation role of *gntR* gene in *Pseudoalteromonas* sp. Rf-1 for LAAO biosynthesis. In addition, our data showed that its disruption only caused slight increase of *lao* gene expression. This could suggest that the *gntR* gene probably downregulates some genes at transcriptional level which in turn regulate LAAO production at posttranscriptional level. However, this postulation remains to be further determined.

On the other hand, we identified 7 possible genes that are likely involved in upregulation pathway of LAAO activity. Three of proposed genes coding for TonB-dependent heme receptor family protein (TdhR), non-ribosomal peptide synthetase (NrpS) and methylase led to decreased LAAO activity after mutation. TdhR is predicted to localize at bacterial membrane, and can sense signals from extracellular or intracellular messengers and transfer them across membrane, leading to transcriptional activation of target genes [37]. The disruption of this gene resulted in extremely significant (P<0.001) decrease of *lao* gene expression, thus suggesting that the *tdhR* gene in *Pseudoalteromonas* sp. Rf-1 may positively regulate LAAO biosynthesis at transcriptional level. Moreover, TdhR protein can interact with outer membrane receptor that can carry specific substrates into cytoplasm space [37]. Thus, it is possible that TdhR may also be involved in absorption of substrates for LAAO biosynthesis. The NrpS protein is generally responsible for introduction of amino acid, and can control the production level of the corresponding peptides. Besides, NrpS also has formyltransferase activity and hydroxymethytransferase activity. Therefore, the NrpS protein may affect the substrate absorption for LAAO biosynthesis and posttranscriptional modification of LAAO. Moreover, our data indicated that the disruption of *nrpS* gene extremely significantly (P<0.001) decreased *lao* gene expression, suggesting that *nrpS* gene in *Pseudoalteromonas* sp. Rf-1 probably participates in upregulation of LAAO biosynthesis at transcriptional level, too. Similarly, considering that the insertion of transposon into methylase gene in mutant B20 resulted in both extremely significant (P<0.001) decrease of *lao* gene expression and extremely significant (P<0.001) decrease of LAAO activity, we postulated that this gene is potentially involved in upregulation of LAAO biosynthesis at transcriptional level.

Finally, we identified 4 upregulating genes that would abolish LAAO activity if disrupted. The disrupted gene in mutant B19 probably codes for Na^+^/H^+^ antiporter NhaD and related arsenite permease (NhaD). NhaD is a ubiquitous protein usually in cytoplasmic membrane and in membranes of many organelles, and plays a primary role in homeostatic mechanisms and transmembrane transport of substances, such as H_2_O_2_, protein and vitamins. It is proposed that the disruption of this gene may disturb LAAO secretion. In addition, the disruption of this gene caused significant decrease (P<0.05) of *lao* gene expression, thus suggesting that the *nhaD* gene in *Pseudoalteromonas* sp. Rf-1 may also indirectly upregulate LAAO biosynthesis at transcriptional level. The disrupted genes in mutants B12 and B1 matched with the ones encoding N-acetyltransferase GCN5 (NaT5) and SAM-dependent methytransferase (SdmT), respectively. These two proteins are responsible for acetylation of Lys and Cys residues, and methylation of Glu, His, Lys and Arg residues, respectively, both participating in posttranslational modification of proteins [38]. These amino acid residues account for a huge amount of amino acids in LAAO of *Pseudoalteromonas* sp. Rf-1 (data not shown). Thus, NaT5 and SdmT may play a role in posttranslational modification of LAAO. Besides, NaT5 is responsible for acetylating the wobble base of elongator tRNA^Met^ by utilizing acetyl-coenzyme A (CoA) and ATP (or GTP) to form N^4^-acetylcytidine (ac4C) [39]. The ac4C formation at wobble base of elongator tRNA^Met^ is thought to ensure the precise recognition of AUG codon by preventing misreading of near-cognate AUA codon [39, 40], thus ensuring the correct initiation of protein translation of protein. SdmT can catalyze 2’-O-methylation of cytidine 1402 and N^4^-methylation of cytidine 1402 in 16S rRNA. It has been found that methylation modification in 16S rRNA is necessary for stringent selection of the initiator tRNA and efficient translation initiation at UUG and GUG [41, 42]. All these suggest that both *nat5* and *sdmT* genes in *Pseudoalteromonas* sp. Rf-1 may positively regulate the translation initiation of LAAO as well. Considering the fact that the disruption of these two genes extremely significantly (P<0.001) downregulated *lao* gene expression, it is clear that both genes are probably involved in positive regulation on LAAO biosynthesis also at transcriptional level. The disrupted gene in another mutant B6 with no LAAO-activity matched with the one coding for ketol-acid reductoisomerase (KarI). This enzyme can catalyze conversion of acetohydroxy acids into dihydroxy valerates, which is a synthetic pathway of the essential branched side chain of amine acids Val and Ile. Probably, the disruption of *karI* gene in *Pseudoalteromonas* sp. Rf-1 will affect the synthesis of amino acids Val and Ile in LAAO, thus leading to loss of LAAO activity. Since the *karI* gene disruption extremely significantly (P<0.001) downregulated *lao* gene expression, it is clear that the *karI* gene positively regulates LAAO biosynthesis also at transcriptional level.

To our best knowledge, it is the first time to explore many genes involved in regulation of LAAO activity in *Pseudoalteromonas* sp. Rf-1 at transcriptional, posttranscriptional, translational and/or posttranslational level. Although western blot and/or site-directed mutation will be desired to confirm the molecular function of the explored genes in LAAO biosynthesis, our findings pave a road for exploiting the mechanisms underlying LAAO biosynthesis and secretion, and provide the understanding of biological and ecological roles in *Pseudoalteromonas* sp. Rf-1 by controlling LAAO activity in marine environment.

## Materials and Methods

### Strains, plasmids and media

All bacterial strains and plasmids used in this study were shown in Table 3. *Pseudoalteromonas* sp. Rf-1 (Rif^r^) was grown in a marine medium (MM) (0.3% yeast extract, 0.5% peptone, 3% sea salt (Sigma)) at 28°C with a shaking of 120 rpm. All *E*. *coli* cells were cultured in Luria-Bertani (LB) medium at 37°C with a shaking of 150 rpm. Unless otherwise specified, to make solid medium, agar was added to a final concentration of 2%. When required, antibiotics were added to medium with final concentrations as below: rifampicin (Rif) 50 μg/ml, ampicillin (Ap) 100 μg/ml, gentamicin (Gm) 50 μg/ml, streptromycin (Str) 50 μg/ml and kanamycin (Km) 50 μg/ml.

**Table 3:** Bacterial strains and plasmids used in this study.

Strain or plasmid	Description and/or relevant genotype	Reference or source
Strains		
*E*. *coli*		
S17–1(λpir)	Tp^r^ Sm^r^, *recA ths hsdRM* ^+^, λpir phage lysogen RP4::Mu::Km Tn*7*	[23, 25]
DH5α	F^-^, φ 80d*lacZ* ΔM15, Δ(*lacZYA*-*argF*) U169, *deoR*, *recA1*, *endA1*, *hsdR17* (*rK* ^-^, *mK* ^+^), *phoA*, *supE44*, *λ* ^-^, *thi-1*, *gyrA96*, *relA1*; Host of plasmid pRK2013	Our lab
HB101	*supE*44, △ (*mcr*C-*mrr*), *recA*13, *ara*-14, *proA*2, *lacY*1, *galK*2, *rpsL*20, *xyl*-5, *mtl*-1, *leu*B6, *thi*-1; host of plasmid pBBR1MCS-5	Our lab
*Pseudoalteromonas* sp.		
Rf-1	Wild type; spontaneously resistant to rifampicin	This study
B3	mutant with increased LAAO activity; Rif^r^, Km^r^	This study
B21	mutant with increased LAAO activity; Rif^r^, Km^r^	This study
B22	mutant with increased LAAO activity; Rif^r^, Km^r^	This study
B9	mutant with reduced LAAO activity; Rif^r^, Km^r^	This study
B10	mutant with reduced LAAO activity; Rif^r^, Km^r^	This study
B11	mutant with reduced LAAO activity; Rif^r^, Km^r^	This study
B17	mutant with reduced LAAO activity; Rif^r^, Km^r^	This study
B20	mutant with reduced LAAO activity; Rif^r^, Km^r^	This study
B1	LAAO-deficient mutant ; Rif^r^, Km^r^	This study
B6	LAAO-deficient mutant; Rif^r^, Km^r^	This study
B12	LAAO-deficient mutant; Rif^r^, Km^r^	This study
B19	LAAO-deficient mutant; Rif^r^, Km^r^	This study
Plasmids		
pLOF/Km	Ori R6K, mob RP4, Ap^r^, mini-Tn*10* Km^r^; delivery vector	[24]
pBBR1MCS-5	Rep, LacZ, Gm^r^; delivery vector for complementation	[28]
pRK2013	Derivative of IncP-1 plasmid RK2, *Tra* ^+^, ColE1, oriT, Km^r^; assistant plasmid for complementation	[29]

### Conjugation and transposon mutagenesis

To generate mutants with altered LAAO activity, transposon mutagenesis [23, 25] was performed in rifampicin-resistant *Pseudoalteromonas* sp. Rf-1. In brief, the strain Rf-1 was inoculated into MM with 50 μg/ml Rif and the *E*. *coli* S17-1(λpir) harboring suicide vector pLOF/Km inoculated into LB (0.5% NaCl) with 100 μg/ml Ap and 50 μg/ml Km. After overnight incubation, both strains were reinoculated into their own fresh medium without antibiotics and allowed to reach exponential growth phase. To transfer the mini-Tn*10* transposon together with kanamycin resistance marker on pLOF/Km to strain Rf-1 cells through conjugation, a 40 μl sample of the exponentially growing recipient Rf-1 cells was spotted onto the surface of conjugation medium (LB-MM, obtained by mixing equal amounts of the two media) agar plate and allowed to dry before another 40 μl of *E*. *coli* donor cells were added to the top of previous spot. Controls with only strain Rf-1 or *E*. *coli* were also prepared. After overnight conjugation, the cells were then collected by scraping and suspended in 1 ml of MM. After appropriate dilution, the cells were plated onto selective MM with 50 μg/ml Rif and 50 μg/ml Km, and incubated at 28°C for 48 h. Finally, the target mutants with altered LAAO activity were screened using Prussian blue agar assay method [22].

### Prussian blue agar assay for screening of mutants with altered LAAO activity

Mutants with deficient or altered LAAO activity were selected based on high throughput in-gel determination method of Prussian blue agar assay [22]. In brief, individual mutant was grown in MM with Rif and Km for 2 d at 28°C with a shaking of 160 rpm. After measurement of absorbance reading (600 nm) and adjustment of cell density if necessary, 1 ml of the above culture was added to 250 ml conical flask containing 50 ml of MM with Rif and Km for fermentation at 28°C with shaking at 160 rpm. After 72-h fermentation, the culture supernatant was harvested with a centrifugation at 5 000 rcf (relative centrifugal force) for 10 min at 4°C. Then, the Prussian blue agar assay plate containing 1.0 g/l FeCl_3_·6H_2_O, 1.0 g/l potassium hexacyanoferrate (III), 5 mM L-Leu and 20 g /l agar with pH of 7.5 was fabricated and small wells on plate were made using a puncher with a diameter of 6 mm. Next, 50 μl of culture supernatant was subjected to each well on assay plate. After incubation at room temperature for 1 h, LAAO activity was determined based on the diameter of the formed Prussian blue halo [22]. For each experiment, triplicate reactions were performed. All Data are presented as mean ± standard error of the mean. Significance of mean difference of LAAO activity was statistically analyzed by ANOVA.

### Determination of transposon-inserted gene

Genomic DNAs were extracted from the transposon-inserted mutants of strain Rf-1 using a bacterial genomic DNA extraction kit (GE, USA). The DNA sequence flanking the inserted transposon in a mutant was determined using high-efficiency thermal asymmetric interlaced PCR (hiTAIL-PCR) [27] (first time). The primers used for hiTAIL-PCR were shown in Table 4. In the first round, PCR was performed in 20 μl of reaction mixture containing 2.0 μl of PCR buffer, 200 μM dNTPs, 1.0 μM of any one of the LAD primers [27], 50 nM DTn10AP1, 2.5 U rTaq polymerase (TaKaRa, Dalian, China), and 20–30 ng DNA. Then, a secondary round of PCR was employed in 50 μl of reaction mixture containing 1.0 μl of 10-fold diluted first-round PCR product, 1.0 μM of any one of the LAD primers, 50 nM DTn10AP2 and 2.5 U rTaq polymerase. Finally, a third round PCR was conducted in 50 μl of reaction mixture consisting of 1.0 μl of 10-fold diluted second-round PCR product, 1.0 μM of any one of the LAD primers, 50 nM DTn10AP3 and 2.5 U rTaq polymerase. All the thermal conditions were shown in Table 5. The PCR products from the second or third round of PCR amplification were electrophoresed on a 1.5% agarose gel and the desired amplicons were purified using a gel extraction kit (Qiagen, CA, USA) for TA cloning with pMD19-T simple vector (TaKaRa, Dalian, China). After sequencing by Sangon Biotech (Shanghai, China), the intermediate sequences of disrupted genes in mutants were collected and compared with the relevant genes in the GenBank of NCBI, EMBI databases and/or Prosite.

**Table 4:** Primers used in high-efficiency thermal asymmetric interlaced PCR.

primers^a^	Oligonucleotide sequences^b^
DTn10AP1	5’-TTGCCCGACATTATCGCGAGCCCAT-3’
DTn10AP2	5’-CAACACCTTCTTCACGAGGCAGACC-3’
DTn10AP3	5’-CGTTGCGCTGCCCGGATTACAGCCG-3’
LAD1-1	5’-ACGATGGACTCCAGAGCGGCCCGCVNVNNNGGAA-3’
LAD1-2	5’-ACGATGGACTCCAGAGCGGCCCGCBNBNNNGGTT-3’
LAD1-3	5’-ACGATGGACTCCAGAGCGGCCCGCHNVNNNCCAC-3’
LAD1-4	5’-ACGATGGACTCCAGAGCGGCCCGCVVNVNNNCCAA-3’
LAD1-5	5’-ACGATGGACTCCAGAGCGGCCCGCBDNBNNNCGGT-3’
Fsdmt1	5’-CATATTGTGTCTGAAACTTTGGTCC-3’
Fsdmt2	5’-TACCTCACCCAGCAACAAGGACTTT-3’
Fsdmt3	5’-CACCTCACCCAGCAACAAGGACTTT-3’
RnhaD1	5’-CAATTACCTGGTGCCGGCGGTCATT-3’
RnhaD2	5’-ACTTTTCCTGATCACCATTGCCACC-3’
RnhaD3	5’-GTCACAGTTTGCTACATTTGCCACC-3’
FnhaD1	5’-CATCCATACGCGCAAGGCATCGAAT-3’
FnhaD2	5’-AGGCATGCTCTGTCAGCTTGGGGAT-3’
FnhaD3	5’-AGATCAACCCGGCCGCCACTAGTAC-3’
Rkari1	5’-CGAAGAGCTCAAGGCCATTATGCGC-3’
Rkari2	5’-GAAGACGATGCAAAACTGCTAAGCT-3’
Rkari3	5’-GAGCAGGAGTTTTTTGACAATGGTA-3’
Fkari1	5’-GTGTGGCGCCTTGCTTCATTAGCGG-3’
Fkari2	5’-GTTTAGTACCACATCAGCCTGGTGG-3’
Fkari3	5’-CCAGAATCACGTAAGTTAAGACCCT-3’

^a^ DTn10AP1, DTn10AP2 and DTn10AP3 are primers specific to transposon gene and were used to retrieve the disrupted genes in mutants; LAD1-1, LAD1-2, LAD1-3, LAD1-4 and LAD1-5 are arbitrary primers which were designed based on the report [27]; the remaining primers were designed based on the intermediate sequences of the disrupted genes in mutants B1, B6 and B19, respectively, and used to amplify for 3’-region of *sdmT* gene (Fsdmt1 (1st), Fsdmt2 (2nd), Fsdmt3 (3rd)), 5’-region of *nhaD* gene (RnhaD1 (1st), RnhaD2 (2nd), RnhaD3 (3rd)), 3’-region of *nhaD* gene (FnhaD1 (1st), FnhaD2 (2nd), FnhaD3 (3rd)), 5’-region of *karI* gene (Rkari1 (1st), Rkari2 (2nd), Rkari3 (3rd)), and 3’-region of *karI* gene (Fkari1 (1st), Fkari2 (2nd), Fkari3 (3rd)).

^b^ V = A/C/G; N = A/C/G/T; B = C/G/T; H = A/C/T; D = A/G/T.

**Table 5:** Thermal Conditions in hiTAIL-PCR.

First TAIL-PCR		Time	Secondary TAIL-PCR		Time	Third TAIL-PCR		Time
Step	Temperature (°C)		Step	Temperature (°C)		Step	Temperature (°C)	
1	93	2:00	1	94	0:22	1	94	0:20
2	95	1:00	2	55	1:00	2	68	1:00
3	94	0:30	3	72	3:00	3	72	3:00
4	60	1:00	4	Go to step 1	1 cycle	4	94	0:30
5	72	3:00	5	94	0:20	5	68	1:00
6	Go to step 3	10 cycles	6	60	1:00	6	72	3:00
7	94	0:30	7	72	3:00	7	94	0:30
8	25	2:00	8	94	0:20	8	50	1:00
9	Ramping to 72	0.5°C/s	9	60	1:00	9	72	3:00
10	72	3:00	10	72	3:00	10	Go to step 1	10 cycles
11	94	0:20	11	94	0:20	11	72	10:00
12	58	1:00	12	44	1:00	12	End	
13	72	3:00	13	72	3:00	13		
14	Go to step 11	25 cycles	14	Go to 5	13 cycles			
15	72	5:00	15	72	5:00			
16	End		16	End				

To retrieve the entire length of *sdmT*, *karI* and *nhaD* gene sequences from LAAO-deficient mutants B1, B6 and B19, respectively, the hiTAIL-PCR was employed again (second time) to amplify the 5’- and 3’-regions of *sdmT*, *karI* and *nhaD* genes. As shown in Table 2, specific primer sets were designed based on the intermediate region collected from the first time of hiTAIL-PCR. After hiTAIL-PCR (Table 3), the desired products were recovered from agarose with gel extraction kit for TA cloning. After sequencing, the contigs of 5’-region, intermediate region, and 3’-region were assembled for analysis.

### Complementation of the disrupted genes through tri-parental mating

To complement the disrupted genes of *sdmT*, *karI* and *nhaD* in three mutants of B1, B6 and B19, respectively, the entire three genes of *sdmT*, *karI* and *nhaD* were individually amplified using three primer pairs (Table 6), SdmtF and SdmtR, KariF and KariR, and NhaDF and NhaDR, respectively. The *Pfu* DNA polymerase (Promega, USA) was used to amplify 594 bp of *sdmT* gene, 1473 bp of *karI* gene and 1455 bp of *nhaD* gene. After addition of 3’-adenine overhang by *Taq* DNA polymerase (TaKaRa, Dalian, China), the desired PCR product was gel-purified and cloned into pMD19-T simple vector. After digestion with *Hin*d III/*Eco*R I, *Kpn* I/*Hin*d III and *Kpn* I/*Xba* I, the *sdmT*-gene, *karI*-gene and *nhaD*-gene fragments were individually cloned into expression vector pBBR1MCS-5 [28] to yield recombinant plasmids pBBR1MCS-5/*sdmT*, pBBR1MCS-5/*karI* and pBBR1MCS-5/*nhaD*, respectively, and separately transformed into *E*. *coli* HB101. Then, with assistance of plasmid pBR2013 [29] in *E*. *coli* DH5α, these three constructed plasmids were introduced into mutants B1, B6 and B19, respectively, for complementation of the corresponding disrupted gene into each mutant cell through tri-parental conjugation which was performed as below.

**Table 6:** Primers for the amplification of full length genes of *sdmt*, *kari* and *nhad*.

Primers	Oligonucleotide sequences	Restriction sites^b^
SdmtF^a^	5’-CCAAGCTTGGATGGATAAACCTTACTCTC-3’	*Hin*d III
SdmtR	5’-CGGAATTCCGTTACTGCCGTTTAAAAATC-3’	*Eco*R I
KariF	5’- GGGGTACCCCATGGCGAACTATTTCAATT-3’	*Kpn* I
KariR	5’-CCAAGCTTGGTTATATTATTTTCTTCATC-3’	*Hin*d III
NhaDF	5’-GGGGTACCCCATGAAAAACTCGCTATATGT-3’	*Kpn* I
NhaDR	5’-GCTCTAGAGCTCAAACACAGTGAATAGCTC-3’	*Xba* I

^a^ “F” refers to forward primer and “R” to reverse one;

^b^ Restriction sites are underlines.

First, the mutant of strain Rf-1 (receptor), *E*. *coli* HB101 with corresponding recombinant plasmid (donor) and *E*. *coli* DH5α with pBR2013 (assistor) were separately inoculated into MM with 50 μg/ml Rif and 50 μg/mL Km, LB with 50 μg/ml Gm and LB with 50 μg/ml Km, respectively, and allowed to reach exponential growth phase. Then, 2 ml of each culture was centrifugated at 2 000 rcf for 5 min to collect the cells. After washing once, each cell pellet was resuspended in 0.5 ml of conjugation medium (mixture of 1/2 MM and 1/2 LB) and three resuspensions were mixed together for another centrifugation at 5 000 rcf for 5 min. After removal of supernatant, the cell mixture of mutant, HB101 and DH5α was resuspended in 0.2 ml of conjugation medium and dropped to sterilized microporous membrane (size diameter 1 cm; aperture 0.45 μm) on conjugation medium plate for overnight conjugation at 30°C. Finally, the microporous membrane was transferred to 3 ml of MM for washing with vortex. After appropriate dilution, 50 μl of cell solution was spread on MM with 50 μg/ml Rif, 50 μg/ml Km and 50 μg/ml Gm. After incubation at 30°C, the target colonies were confirmed with molecular analysis and used for detection of LAAO activity and *lao* gene expression.

### Analysis of lao gene expression by qRT-PCR

To measure the *lao* gene (access no. KP165133 in GenBank) expression, qRT-PCR [26] was used to amplify cDNA products reversely transcribed from mRNA of mutants selected. In brief, individual mutant was grown in MM with Rif and Km for 2 d at 28°C with a shaking of 160 rpm. Next, after measurement of absorbance reading (600 nm) and adjustment of cell density if necessary, 1 ml of the above culture was added to 250 ml conical flask containing 50 ml of MM with Rif and Km for incubation at 28°C with shaking at 160 rpm. After 72-h cultivation, the culture supernatant was harvested with a centrifugation at 5 000 rcf for 5 min at 4°C. Then, the total RNA was extracted using an RNAiso Plus (Total RNA extraction reagent) kit (Sangon, Shanghai, China). RNA integrity was determined based on OD_260 nm_/OD_280 nm_ ratio (>1.95), and 500 ng of DNA-free RNA with high-quality was reversely transcribed to cDNA in a 10 μl volume using PrimeScript RT Master Mix (Perfect Real Time) kit. After appropriate dilution, the cDNA was used for amplification of 108 bp *lao* gene fragment with primer set of laoF (5’-ATACGCCAAGTGCCTCAGTG-3’) and laoR (5’-TCCGTCAGCCCGTTAAAGTC-3’) by using the SYBR green (Tli RNaseH Plus) kit. The PCR was run on CFX Connect Real-Time System (Bio-Rad, Hercules, CA) with an amplification protocol consisting of an initial denaturation at 95°C for 10 min, followed by 40 cycles of denaturation at 95°C for 15 s and annealing/elongation at 60°C for 30 s. Immediately after the final PCR cycle, a melting-curve analysis was made to determine the reaction specificity based on the observation of melting temperature from product. Unless otherwise specified, all the kits above were purchased from TaKaRa (Dalian, China).

The cycle threshold (*C*_T_) for each PCR was determined using STATVIEW software which automatically set the threshold signal at the log phase of the amplification curve. Several dilutions of each cDNA sample were assayed for gene of interest in order to obtain a linear regression between *C*_*T*_ value (ranging from 10 to 30 cycles) and log of the cDNA. The amplification efficiency of gene was retrieved from the slope of that linear regression according to the formula *E* = 10^(−1/slope)^. The 116 bp of housekeeping 16S rRNA gene fragment (access no. KP234252 in GenBank) was amplified using primer set of 16SF (5’-AAGAAGCACCGGCTAACTCC-3’) and 16SR (5’-TCTCGCTTAATCAACCGCCT-3’) and treated as the internal control to verify that there were equal amounts of target cDNA in all samples. The relative expression (RE) of target gene (TG) in mutant or complon compared to that in wild type Rf-1 (WT) was calculated by the comparative *C*_T_ method [43]. In brief, the difference of *C*_T_ between TG and reference 16S rRNA gene (RG) was calculated according to the formula:

$${\Delta C}_{\text{T}(\text{TG})}={\text{C}}_{\text{T}(\text{TG})}-{\text{C}}_{\text{T}(\text{RG})}$$

Then, the RE of TG compared to RG was obtained by the equation:

$$\text{RE}=2^{-{\Delta C}_{\text{T(TG)}}}$$

Finally, the RE of TG in mutant or complon compared to that in WT was achieved as below:

$$\text{RE}=2^{-{\Delta C}_{\text{T(TG, Mutant or Complon)}}}/2^{-{\Delta C}_{\text{T(TG, WT)}}}$$

For each experiment, triplicate reactions were performed. All Data are presented as mean ± standard error of the mean. Significance of mean difference of relative gene expression was statistically analyzed by ANOVA.

## Supporting Information

S1 Dataset Expression level of 16S rRNA gene and *lao* in mutants and wild type strain Rf-1. (XLS)

S2 Dataset Statistical analysis of mean difference in relative expression of *lao* between mutant and wild type strain Rf-1 by ANOVA. (TXT)

S3 Dataset Expression level of 16S rRNA gene and *lao* in mutants, complons and wild type strain Rf-1. (XLS)

S4 Dataset Statistical analysis of mean difference in relative expression of *lao* between mutant and wild type strain Rf-1 or complon by ANOVA. (TXT)
